# Exploring Punicalagin Potential Against Cancers: A Comprehensive Review

**DOI:** 10.1002/fsn3.70072

**Published:** 2025-02-27

**Authors:** Muhammad Hammad Ul Hassan, Muhammad Shahbaz, Ushna Momal, Hammad Naeem, Muhammad Imran, Mohamed A. Abdelgawad, Mohammed M. Ghoneim, Ehab M. Mostafa, Ahmed H. El‐Ghorab, Suliman A. Alsagaby, Waleed Al Abdulmonem, Muzzamal Hussain, Tadesse Fenta Yehuala

**Affiliations:** ^1^ Department of Food Science and Technology Muhammad Nawaz Shareef University of Agriculture Multan Multan Pakistan; ^2^ Department of Food Science and Technology University of Narowal Pakistan; ^3^ Department of Pharmaceutical Chemistry College of Pharmacy, Jouf University Sakaka Aljouf Saudi Arabia; ^4^ Department of Pharmacy Practice College of Pharmacy, AlMaarefa University Riyadh Saudi Arabia; ^5^ Department of Pharmacognosy College of Pharmacy, Jouf University Saudi Arabia; ^6^ Pharmacognosy and Medicinal Plants Department Faculty of Pharmacy (Boys), Al‐Azhar University Cairo Egypt; ^7^ Department of Chemistry College of Science, Jouf University Saudi Arabia; ^8^ Department of Medical Laboratory Sciences College of Applied Medical Sciences, Majmaah University Saudi Arabia; ^9^ Department of Pathology College of Medicine, Qassim University Buraidah Kingdom of Saudi Arabia; ^10^ Department of Food Sciences Government College University Faisalabad Faisalabad Pakistan; ^11^ Faculty of Chemical and Food Engineering, Bahir Dar Institute of Technology, Bahir Dar University Ethiopia

**Keywords:** androgen receptor, anticancer, antitumor, cancers, cell proliferation, colon cancer, phytochemical, punicalagin

## Abstract

Punicalagin, being a bioactive polyphenol, has gained significant interest owing to its potential anticancer effects. Researchers are studying punicalagin to determine its ability to inhibit cancer cell proliferation. This paper focuses on highlighting the therapeutic potential of punicalagin against tumors and cancers. Punicalagin inhibits tumor growth within the body through various cellular pathway interactions. This compound effectively eliminates tumor cells from the liver, stomach, prostate, and lungs. Different animal model studies have demonstrated the potential of punicalagin in blocking the MAPK/ERK and PI3K/AKT/mTOR signaling pathways, fighting against cancer. Through the inhibition of cell division, punicalagin may be capable of eradicating breast cancer cells. Punicalagin strongly inhibits the activities of vascular endothelial growth factor (VEGF), matrix metalloproteinase‐9 (MMP‐9), and uPA. Punicalagin effectively inhibits the androgen receptor (AR), a protein essential for the development and metastasis of prostate cancer. Lung cancer cells can be eliminated by initiating the caspase cascade and inhibiting protein synthesis, which is facilitated by punicalagin. In numerous animal models, punicalagin significantly reduces metastasis. Cyclokinase B1 and cyclin‐dependent kinase 2 promote colon cancer; when colon cancer cells reach the G2/M phase, punicalagin induces apoptosis by inhibiting the action of these proteins. Punicalagin inhibits tumor development and cancer propagation by inhibiting blood vessel proliferation. There is still a need for further clinical trials and studies to fully reveal punicalagin's potential as well as its safety; despite the fact that it may decrease the threat of increasing tumors and provide an alternative treatment for many cancers.

## Graphical abstract

This paper focuses on highlighting the therapeutic potential of punicalagin against tumors and cancers. Punicalagin inhibits tumor growth within the body through various cellular pathway interactions. This compound has efficacy in eliminating tumor cells from the liver, stomach, prostate, and lungs. Different animal model studies have demonstrated the potential of punicalagin in blocking the MAPK/ERK and PI3K/AKT/mTOR signaling pathways, fighting against cancer. Through the inhibition of cell division, punicalagin may be capable of eradicating breast cancer cells. Punicalagin strongly inhibits the activities of vascular endothelial growth factor (VEGF), matrix metalloproteinase‐9 (MMP‐9), and uPA. Punicalagin effectively inhibits androgen receptor (AR), a protein essential for the development and metastasis of prostate cancer.

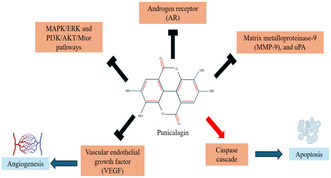

## 1 Introduction

**FIGURE 1:**
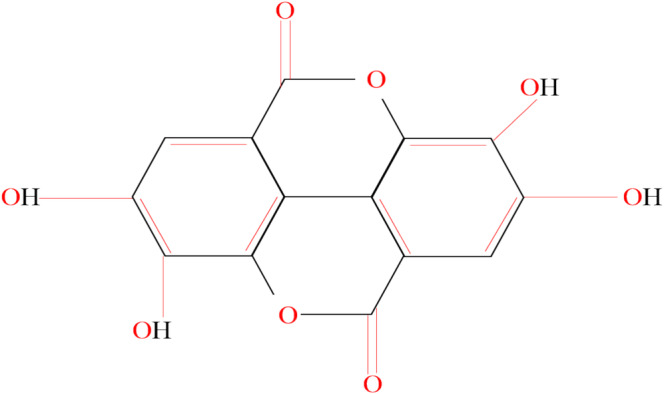
Chemical structure of punicalagin. This figure highlights the distinct framework with glucose moieties and gallic acid derivatives. The detailed molecular structure emphasizes the functional groups contributing to its biological activities. AKT, AK strain transforming; HGFR, hepatocyte growth factor receptor; HMMR, hyaluronan‐mediated motility receptor; NF‐kB, nuclear factor kappa‐light‐chain‐enhancer of activated B cells; VEGF, vascular endothelial growth factor.

The bioactive polyphenol, punicalagin (2,3‐hexahydroxydiphenoylgallagyl‐D‐glucose), can be extracted from *Punica granatum L*. It exhibits a high bioavailability and is the largest ellagitannin molecule soluble in water. Pomegranate juice, extract, and pomegranate peel extract are all rich in punicalagin (Subkorn et al. 2021). Previous studies have investigated the therapeutic properties of punicalagin on a diverse range of tumor cells. Promyelocytic leukemia cells, colon cancer lines, and glioma cells were recognized to have been stimulated to undergo apoptosis under the influence of punicalagin. This involved controlling cell proliferation and apoptosis in addition to influencing cell cycle proteins such as Bcl‐2, Bax, and the Bcl‐2‐linked death promoter (Zhang et al. 2020). The chemical structure of punicalagin is shown in Figure 1.

Punicalagin (C_48_H_28_O_30_), which is mainly present in pomegranate skin and juice, is one of several polyphenols and antioxidants recognized as inhibitors of cancers (Subkorn et al. 2021). The concentration of punicalagin in pomegranate skin varies from 10 to 50 mg/g—the highest among common fruits. It is amorphous, brownish‐yellow, and highly polar; it dissolves in many organic solvents, including ethanol, methanol, water, or acetonitrile. Among the phenolic compounds, this substance has more antioxidant potential than those remaining despite having only 3–4 phenolic hydroxyl groups per molecule. The α/β punicalagin ratios rose between pH values of 2.2 and 3.5, reaching a peak at a pH value of 3.5. However, ratios fell off dramatically as the pH went from 3.5 to 8 (Chen et al. 2023). The hydroxyl radical and the superoxide radical are two extremely reactive compounds, also known simply as radicals, because they contain unpaired electrons. In the course of regular metabolic processes, radicals are produced in all living things through oxidation reactions. When unregulated, the concentration of free radicals can increase in response to stimuli, including environmental stress, injury, and pathogen harm. Compared to the unsaturated lipids found in cell membranes, free radicals are highly reactive and can cause damage to any biomolecule, including proteins, carbohydrates, DNA, and lipids. This harmful impact has the potential to cause multiple diseases, including inflammation, cancer, and coronary heart disease (Aloqbi et al. 2016).

Punicalagin is widely used in the treatment of a wide range of medical conditions, covering but not restricted to diabetes, vaginitis, cancers, high blood pressure, tooth decay, hyperlipidemia, autoimmune diseases, constipation, bronchitis, cough, hemophilia, male infertility, cardiovascular disease, acquired immunodeficiency syndrome (AIDS), asthma, oral tumors, ulcers, dementia, and malaria. The numerous pharmacological effects of pomegranate polyphenols are incredible, especially punicalagin. These compounds have been found to possess an extensive variety of therapeutic properties, such as anticoagulants, liver‐protective, antigenotoxic, and anti‐inflammatory activities. Their ability to exert such a wide range of beneficial effects is evidence of the immense potential of these natural substances. Through their complex mechanisms of action, pomegranate polyphenols hold enormous potential in the field of medicine and health (Benchagra et al. 2021). Cancer is a genetic disease caused by changes in the information carried in DNA, which serves as cellular hereditary material; this leads to abnormal patterns of gene expression. Among the various modes of cell death, programmed ones include apoptosis, autophagy, and necrosis (Kilit and Aydemir 2023a). Punicalagin has been shown to prevent cancer cell proliferation and promote apoptosis through different mechanisms, such as regulating the pathways involved in oxidative damage and inflammation (Khwairakpam et al. 2018). Punicalagin can stop the growth and reduce the viability of cancer cells by changing their signal transduction pathways into the cell cycle (Yasmin et al. 2023; Xie et al. 2022). Several studies have found that punicalagin has the potential to fight against tumors such as leukemia, osteosarcoma, and various cancers (Sun et al. 2023).

## 2 Methodology

For this review, papers were selected on the basis of year of publication and not older than 8 years, with very few older than 8 years owing to their high relevancy and importance to the subject. Papers relating to drug formulations, drug application, and drug delivery using punicalagin were excluded. The papers reporting the main effects of punicalagin on cancer and describing the underlying pathways were included. The largest database, Google Scholar, was primarily used. The main gap in the existing literature is the absence of collected data on the main anticancer effects of punicalagin as per recent literature. This paper includes recently revealed mechanisms of action, potential interactions between different substances, and punicalagin use in particular cancer types. Our paper, being a comprehensive review, provides new insights and increases knowledge of the potential of punicalagin as an anticancer agent by encompassing the most recent discoveries.

## 3 Bioavailability of Punicalagin

Punicalagin is an ellagitannin that is naturally present in the pomegranate skin ( *Punica granatum* ) (Venusova et al. 2021). Although punicalagin has demonstrated potential as a medicine in vitro, the challenge is its inadequate bioavailability in both human and animal models (rats). The factors attributed to the inadequate bioavailability of the substance comprise its substantial molecular weight, which inhibits simple diffusion absorption, and its exceedingly low lipid solubility, which prevents the passage of the bilipid layer covering the gastrointestinal tract (GIT). Furthermore, in healthy humans, the colonic microflora metabolizes punicalagin into hydroxy‐6H‐dibenzopyran‐6‐one derivatives, which are bioavailable but possess relatively low antioxidant activity. To maximize punicalagin's medicinal potential, a solid phase extraction was required to improve its oral bioavailability and biological activity (Vora et al. 2015). The particulate size of punicalagin and ellagic acid (EA) can be reduced, which improves their bioavailability. Common methods for producing nanoparticles include crystallization, hydrated bead milling, high‐pressure homogenization, controlled precipitation, and supercritical fluids. An additional prospective technique to enhance bioavailability involves the encapsulation of punicalagin or ellagic acid within nanoparticles that dissolve naturally (Venusova et al. 2021).

The hydrolysis of punicalagin to ellagic acid in the intestines increases the blood's absorption of this compound. Research indicates that the bacteria in the colon break down ellagic acid into urolithin D. Urolithin C, urolithin A, and urolithin B are produced when urolithin D undergoes further alteration, which causes the development of intermediates with a reduced amount of hydroxyl groups. Absorption into the enterohepatic circulation allows urolithins (6H‐dibenzo [b,d]pyran‐6‐one derivatives) in the form of aglycones or glucuronides to be identified in the bloodstream for a short time before they are eliminated in the urine and feces after 12–56 h. Urolithins have the potential to pass across the blood–brain barrier and exert their anti‐Alzheimer's effect on pomegranate activity (Ramlagan et al. 2022). The research observed that punicalagin and ellagic acid, byproducts of punicalagin hydrolysis, increased levels of liver antioxidant enzymes. The study suggested that punicalagin metabolites can accumulate in various organs, including the prostate and intestine. The application of punicalagin and ellagic acid additionally resulted in a reduction in the amount of cytokines that promote inflammation, specifically TNF‐α, Interferon‐gamma, and IL. There was an associated rise in the absorption rate and the punicalagin concentration. The punicalagin was absorbed into the intestines under the zero‐order kinetic equation (Ramlagan et al. 2022).

## 4 Antioxidant Potential

Punicalagin is a significant antioxidant with great potential to improve our health (Yasmin et al. 2023). It is known that punicalagin, Ellagic acid (EA), and its derivatives have DNA‐protective, antimicrobial, antimutagenic, and antioxidant characteristics. Free radicals are unstable chemicals that can damage cells as well as DNA, which can lead to diseases and cancers; antioxidants protect against these harmful effects. Several modern research studies indicate the antioxidant, free‐radical scavenging properties of punicalagin. In a study, rat liver cells were exposed to an oxidative chemical agent; punicalagin was found to lower lipid peroxidation and oxidative stress in the treated cells. One possible approach to strengthening the brain's defense against oxidative stress (GPx) would be increasing levels of enzymes that neutralize free radicals, such as glutathione peroxidase and superoxide dismutase (SOD), both of which might be positively stimulated by punicalagin. Antioxidant properties define the potential anti‐inflammatory effects of punicalagin. Thus, researchers have established a link between free radicals and inflammation‐induced reactive oxygen species (ROS). Because punicalagin is anti‐inflammatory, there may be an increase in antioxidant capacity as well as a decrease in oxidative stress. The antioxidant effect of pomegranate juice is because it contains hydrolyzed tannins, with punicalagin making up 87% of the observed activity. Its concentration in a liquid can exceed 2 g/L (Yasmin et al. 2023).

Various in vitro studies have been used to examine the antioxidant capacity of punicalagin. Punicalagin has the ability to denature hydroxyl, DPPH, and ABTS radicals. The activity of oxidative stress markers was clearly lower in the liver and kidney muscles in the experimental group. In contrast, antioxidant enzyme activity (such as superoxide dismutase or catalyst) increased considerably. In a clinical trial involving healthy human volunteers, the effects of punicalagin medication on inflammation and oxidative stress were investigated. The participants had taken 500 mg of punicalagin daily over 4 weeks. This indicates that punicalagin medicine may cause health and well‐being benefits by reducing proinflammatory cytokines and also markers of oxidative stress. Investigation into the improvement of cisplatin's chemotherapeutic effect against human cervical cancer cells was conducted. Punicalagin increased the sensitivity of cancer cells, leading to a significant fall in cell viability and an increase in apoptosis (Nasr et al. 2023).

An investigation was conducted to assess the antioxidant potential of punicalagin in animals with chemically induced colon cancer. Punicalagin‐treated rats revealed improved activity of the antioxidant enzymes like superoxide dismutase and catalase, as well as decreased levels of MDA (an index representation for free radicals) in colonic tissues. Punicalagin has also been linked to the cell signaling pathways present in cancer progression and development, as well as an ability to reduce levels of existing cancer cells, stimulate their death, and slow down the speed of demolition to that extent. Whether punicalagin can enhance the effectiveness of other cancer drugs has been discussed. Our study looks at whether punicalagin's antioxidant properties make it possible to prevent breast, prostate, and lung cancers (Cortez‐Trejo et al. 2022).

Researchers, while studying liver cancer, have reported higher levels of punicalagin in animal liver tissues, which indicated higher levels of free radicals markers (MDA) and increased antioxidant enzyme activity (SOD, CAT). Liver cancer cell growth was also suppressed by punicalagin at the same time. Punicalagin may increase the sensitivity of human ovarian cancer cells to paclitaxel, which might improve the drug's capacity to kill these cells. Additionally, the cancer cells' decreased levels of antioxidant stress indicators and reactive oxygen species (ROS) suggested that punicalagin possessed antioxidant properties (Khwairakpam et al. 2018).

### 4.1 Anticancer Perspectives

Punicalagin is gaining popularity as a natural anticancer agent. A quality that contributes significantly to its anticancer potential is its remarkable capacity to stimulate apoptosis. Punicalagin has been shown to inhibit the progression of carcinomas originating from the lung, prostate, breast, and colon. This is accomplished through the induction of cell cycle inhibition and apoptosis, both of which ultimately lead to the failure of tumor cells (Jacob et al. 2018). The proliferation and prevalence of cancer are substantially affected by ongoing inflammation. Therefore, punicalagin is anti‐inflammatory because it can prevent the formation of prostaglandins and cytokines, which are compounds promoting inflammatory reactions. Tumors produce new blood vessels by the process of angiogenesis in order to develop nutrients and oxygen. Punicalagin prevents new blood vessels from forming by inhibiting the production of vascular endothelial growth factor (VEGF), which is critical to angiogenesis. Further studies have discovered that punicalagin makes tumor cells more susceptible to anticancer medications, improving chemotherapy efficiency. It lowers the side effects of chemotherapy by protecting normal cells from harm. Cancer suppressor genes such as p53 and p21, which are critical to inhibiting the spread of cancer cells, have been found to be out‐produced in a way influenced by punicalagin (Venusova et al. 2021).

**FIGURE 2:**
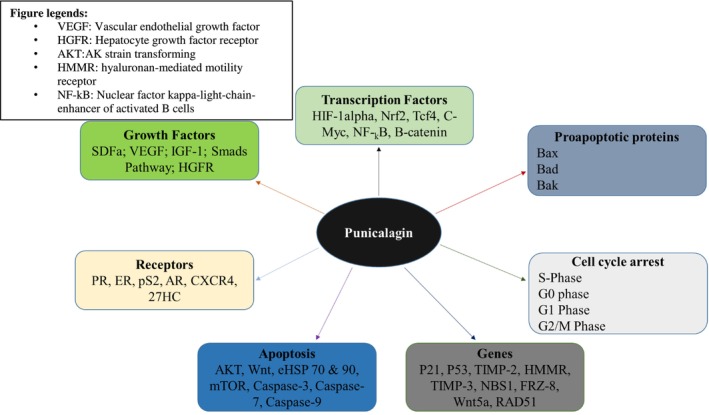
Anticancer factors controlled by punicalagin. A comprehensive diagram showcasing the molecular pathways modulated by punicalagin in cancer. The figure illustrates key factors such as apoptosis induction, cell cycle arrest, angiogenesis inhibition, and metastasis suppression. Specific signaling pathways, including NF‐κB, PI3K/Akt, and MAPK, are highlighted as targets of punicalagin's anticancer effects. IL‐10, interleukin‐10; NF‐kB, Nuclear factor kappa‐light‐chain‐enhancer of activated B cells; ROS, reactive oxygen species; TNF‐α, tumor necrosis factor alpha.

Punicalagin has shown promising anticancer properties, making it a potential agent to be used for tumor prevention and treatment. Cancer cells spread to other organs through the process of metastasis. Punicalagin prevents metastasis by suppressing the activity of enzymes used in cancer cell invasion and dissemination (Usha et al. 2020). In addition, punicalagin has been found to have an influence on different routes for signaling NF‐κB, MAPK/ERK, and PI3K/Akt. Each of these pathways contributes to the development and proliferation of cancer. Punicalagin can prevent the survival and proliferation of cancer cells by modulating these receptor pathways. It is considered that both of these factors contribute to the fundamental igniting condition by causing an imbalance between favorable and provocative intermediates and mitigating others. Macrophages, natural killer cells (NK), and T cells are among the immune cells that have been identified as being stimulated by punicalagin in order to enhance immune function against cancers (Venusova et al. 2021). Damaged organelles and proteins are degraded as part of the cellular process known as autophagy. By maintaining cellular homeostasis, punicalagin inhibits the development of tumors. The anticancer properties of punicalagin can be assigned to its ability to induce autophagy. Punicalagin has been found to exhibit synergistic effects when combined with other anticancer agents. By engaging in cooperative action, these substances can simultaneously enhance their overall anticancer efficacy and reduce their toxicity. Punicalagin is a more favorable choice for tumor prevention and management due to its diverse mechanisms of action. Further investigation and advancement of this polyphenol are needed due to its minimal toxicity and wide availability in various food sources (Berdowska et al. 2021). Anticancer factors controlled by punicalagin are shown in Figure 2.

### 4.2 Punicalagin and Breast Cancer

Breast cancer is the second most prevalent cause of cancer‐related mortality among women of all ages. Moreover, over time, this disease demonstrates considerable heterogeneity between males and females. The rise from 870,000 to around 1,937,000 new cases worldwide among women had an enormous effect on developing countries. As a potential management strategy, prevention of this disease has been the subject of scientific inquiry in recent years. By altering one's diet, an estimated two‐thirds of cancer‐related fatalities could be prevented. These organic constituents are referred to as phytochemicals. Additionally, anticancer properties are present in the pomegranate fruit ( *Punica granatum* ). It has been shown that pomegranate inhibits cellular transformation and proliferation, inflammatory responses, and the angiogenesis process. Furthermore, there has been speculation regarding the potential of its bioactive components to impede the final stages of carcinogenesis and metastasis. There are numerous advantageous impacts of pomegranate peel on breast cancer. The benefits above consist of anti‐estrogenic and anti‐aromatase characteristics, modulation of the transforming growth factor beta (TGF‐β)/Smads pathway activity, suppression of inflammatory cytokines and chemokines, reduction in vascular endothelial growth factor (VEGF) concentrations, downregulation of genes implicated in DNA damage and expression of estrogen‐responsive genes, and disruption of estrogen resistance (Moga et al. 2021). Pancreatic papillary thyroid carcinoma breast cancer cells are subjected to autophagic cell death induced by punicalin via initiation of the MAPK pathway and stopping the mTOR signaling chain (Kilit and Aydemir 2023b).

The mechanism by which punicalagin influences cellular processes in breast cancer was explored in an interesting study at a molecular level. Scientists have used CCK‐8, wound healing, and Transwell assays to study whether or not MCF‐7 and MDA‐MB231 cells migrate, invade, or survive when exposed to varying doses of punicalagin. Later, GOLPH3 (Golgi phosphoprotein 3) was transfected into the cells. The researchers used qRT‐PCR and Western blotting to level the expression of GPLPH3, along with a factor such as E‐Cadherin. Cancer Cell Lines MCF‐7 and MDA‐MB‐231 during treatment with PN at 50 μM or higher dramatically decreased cell viability, migration, and invasion. However, the overstrain of GOLPH3, an enzyme that increased cell viability, motility, and invasion, slightly counterbalanced the detrimental effects of punicalagin therapy on BC cells. The results revealed that punicalagin overstrain of GOLPH3 partially counterbalanced the effects ascribed to PN. The study showed that punicalagin reduced the expression of E‐Cadherin while increasing the expression of MMP‐2, MMP‐9, and N‐Cadherin. As a result, punicalagin provides a potential therapeutic pathway for the management of breast cancer by inhibiting cell viability and metastasis via modulation of GOLPH3 (Pan et al. 2020).

The development and testing of polylactic co‐glycolic acid (PLGA), PLGA‐treated Chitosan (CS), and PLGA‐treated polyethylene glycol (PEG) nano‐prototypes loaded with 5‐fluorouracil (FU) and punicalagin were investigated. The experiment was carried out to identify the toxicological as well as biochemical effects of each nanoformulation. The expression levels of these treatments for apoptosis and survival in cancer cells were then determined at the genomic and protein levels. In vitro experiments with the MDA‐MB‐231 and MCF7 cell lines showed that these new nanoparticles had cytotoxic and apoptotic activity. Also, Bax and Cas‐3 expression levels were elevated. BCL‐2, NF‐kappaB, and PI3k were decreased. Compared to the control group, pungent and nano‐prototypes resulted in much higher levels of zinc (Zn) and nitric oxide (NO). The findings demonstrated that nanoprototypes of PLGA, which were coated in CS and PEG, showed increased effectiveness against cancer by causing cytotoxicity through apoptosis triggered by reactive oxygen species (ROS) (Abd‐Rabou et al. 2022).

Researchers recently investigated the expression of COX‐2, Nrf2, and HSP90 to determine whether pomegranate extract's (PE, rich in punicalagin) anti‐inflammatory mechanism worked during DMBA‐induced breast carcinogenesis in rats. The growth of breast tumors was inhibited depending on the dosage in reaction to PE (0.2–5.0 g/kg), as determined by analyzing samples obtained during a chemopreventive trial. Immunohistochemical methods were used to evaluate the levels of expression for inhibitory B (IB), COX‐2, HSP90, NF‐κB, and Nrf2. Pomegranate extract slowed the degradation of inhibitory IB and blocked the nuclear translocation of nuclear factor B. It also reduced COX‐2 and HSP90 expression while increasing Nrf2 expression and nuclear translocation. During the development of mammary tumors induced by DMBA, these effects were observed. The results suggest that pomegranate extract's PE may inhibit DMBA‐induced mammary carcinogenesis through anti‐inflammatory mechanisms by variably modifying two related molecular pathways, NF‐κB and Nrf2 signaling. Additional studies are required to further characterize the exact modes of action of punicalagin in cancerous cells before in vivo testing with an animal experimental model (Mandal et al. 2017).

### 4.3 Punicalagin and Pancreatic Cancer: A New Approach to Cancer Suppression

In the United States, pancreatic cancer will probably overtake all other cancers as the second most prevalent cause of cancer‐related mortality within the next 2–30 years. It has an approximate 10% 5‐year survival rate in the United States and is a growing cause of cancer‐related mortality. Globally, it ranks seventh in terms of cancer‐related fatalities among both males and females (Mizrahi et al. 2020). PNET, or pancreatic neuroendocrine tumor, originates in the glandular endocrine system. In contrast, acinar cell carcinoma and pancreatic ductal adenocarcinoma (PDAC) are examples of exocrine neoplasms of the pancreas. Pancreatic ductal adenocarcinoma (PDAC) is one of the primary causes of cancer‐related mortality and the most widespread form of pancreatic cancer. In the United Kingdom, < 3% of pancreatic ductal adenocarcinoma PDAC patients have an overall survival of up to 5 years (Lanfredini et al. 2019). Pomegranate juice contains more than 50% of the antioxidants found in the fruit. The high polyphenol content, especially punicalagin, of pomegranate, has been associated with its antioxidant and anti‐atherosclerotic properties (Adaramoye et al. 2017).

In a recent study, the purified punicalagin flavonoid extracted from pomegranate skin was examined in vitro on PANC‐1 (human pancreatic cancer epithelial cell line) cell lines grown under conditions of 5% CO_2_ at 37°C. Therefore, a WST‐1 cell proliferation reagent was used to test the cytotoxic effects of punicalagin for a 24‐h incubation period. Researchers examined whether the cytotoxic effects of punicalagin on cell lines depended upon time and dose. A WST‐1 cell proliferation reagent was used in testing the vitality of cells. The study revealed that punicalagin killed PANC‐1 cancer cell lines (Kilit and Aydemir 2023a). Using a chick chorioallantoic membrane (CAM) model, researchers examined the antiangiogenic effects of pomegranate fruit extract in two cell lines: colon (colo205) and human pancreatic cancer (Suit‐2). DMEM supplemented with 1% penicillin and streptomycin, together with 10% fetal bovine serum, was used. The first experiment involved the use of Matrigel‐encapsulated pancreatic cancer cells. They were treated with PBS, 5 mg/CAM pomegranate for OPC (Suit‐2), 10 mg/CAM pomegranate, and finally with 20 mg/CAM pC (the most). According to the results, Chorioallantoic membrane (CAM) models of pancreatic Suit‐2 showed significantly lower tumor weight and hemoglobin concentration in groups fed with extracts from whole fruit, solution, or filtered liquid. These results suggest that pomegranate extracts, especially when combined with other therapeutic strategies, could hold significant promise as a natural adjunct to chemotherapy, enhancing treatment outcomes for patients with pancreatic cancer (Sudha et al. 2021).

### 4.4 Anticancer Potential of Punicalagin Against Prostate Cancer

Among men, prostate cancer is the second most common tumor since 2018. It was responsible for 358,989 male cancer deaths and 1,276,106 new cases, bringing up 3.8% of all male cancer fatalities. Early‐stage prostate cancer is frequently asymptomatic, progresses slowly, and may necessitate minimal or no treatment. Diet and physical activity significantly influence the development and progression of prostate cancer (Rawla 2019). Numerous studies have provided evidence indicating that extracts derived from pomegranates have anticancer characteristics in various cancer cell lines. These qualities include inhibiting cell proliferation, promoting programmed cell death, preventing cell invasion, and reducing inflammation. Pomegranates comprise flavonoids and other polyphenolic compounds that exhibit significant free radical scavenging and antioxidant capabilities. Pomegranates contain punicalagin (PN) as their most abundant ellagitannin (Adaramoye et al. 2017).

Researchers studied punicalagin's modulatory effects on prostate cancer cell extrinsic apoptotic pathways and chorioallantoic membrane (CAM) angiogenesis. The study was based on the two techniques used in this experiment: 2,2‐diphenyl‐1‐picryhydrazyl (DPPH) radical scavenging test and suppression of lipid peroxidation. A number of different cell lines (BPH‐1, a line from normal prostates; PC‐3 and LNCaP, prostate cancer expressing cells) were grown in culture. They were exposed to PN at concentrations between 10 μM and 100 μM using the XTT colorimetric assay for viability as well as to determine the antiangiogenic effects, a chorioallantoic membrane (CAM) test was employed. Also, DNA fragmentation and enrichment factor (measured by the Cell Death Detection ELISA kit), as well as caspase‐3 and caspase‐8 expression were used to measure apoptosis. The findings indicated that punicalagin (10–200 μM) demonstrated concentration‐dependent inhibition of LPO and a significant DPPH scavenging effect. Furthermore, the inhibitory effect of punicalagin (10–100 μM) on viability in PC‐3 and LNCaP cells was seen to be concentration‐dependent. However, no significant impact on vitality was observed in BPH‐1 cells. Furthermore, the enrichment factors for PC‐3 (2.34 ± 0.05) and LNCaP (2.31 ± 0.26) were seen to be significantly increased by the addition of punicalagin (100 μM) as compared to the control group (1.00 ± 0.00). Furthermore, the reduction of the vessel network in the chorioallantoic membrane (CAM) by punicalagin (50 μM) indicates that it has an anti‐angiogenic effect. Moreover, punicalagin (PN) increased caspase‐3 and caspase‐8 expression in PC‐3. Punicalagin's ability to inhibit cell proliferation in prostate cancer is due to its anti‐angiogenic and apoptotic effects (Adaramoye et al. 2017).

Researchers have also performed clinical trials that may have shown that pomegranate juice slowed the growth of prostate cancer cells. The findings indicate that pomegranate juice constituents, namely luteolin, punicic acid, ellagic acid, and possibly punicalagin, collectively hinder the proliferation of prostate cancer cells, both those dependent on and independent of hormones. Additionally, they hinder the chemotaxis and migration of these cells toward CXCL12, a crucial chemokine in proliferating prostate cancer. A severe combined immunodeficiency mouse model was used to test this theory by administering luciferase‐expressing human prostate cancer cells through the skin around the prostate. Weekly bioluminescence imaging was utilized to track the progression of the tumor. All of the PC‐3 M‐luc tumors failed to metastasize as a result of P, E, and L, preventing the CXCR4/CXCL12 axis from metastasis and primary tumor growth. In addition, luteolin, punicic acid, and ellagic acid significantly prevent the development and spread of very invasive K‐rasG12D prostate cancers. Luteolin, punicic acid, and ellagic acid additionally disrupt preformed endothelial cell (EC) tubes, prevent angiogenesis in vivo, and hinder the adhesion of EC to one another, as evidenced by their ability to prevent human endothelial cell (EC) tube development in culture. Endothelial cells (ECs) are also prone to the suppression of signaling pathways that are activated by angiogenic factors such as interleukin‐8 and vascular endothelial growth factor, by the use of luteolin, punicic acid, and ellagic acid (Wang et al. 2014).

To investigate the antiproliferative and apoptotic effects of pomegranate peel in vivo, another interesting study used a nude mouse model that had human prostate cancer cells (PC‐3) transplanted through the skin. The levels of two cytokines, tumor necrosis factor alpha (TNF‐ α) and vascular endothelial growth factor (VEGF), were measured by an enzyme‐linked immunosorbent assay technique (ELISA). A photodiode array detection device analyzed the active section and reverse‐phase high‐performance liquid chromatography (HPLC) to determine amounts of gallic acid, ellagic acid, and punicalagin. According to the results, in rats carrying tumors, pomegranate peel significantly increased the apoptotic rate and reduced the size of the tumor. The clinical trial showed that after administration of pomegranate peel, the amounts of vascular endothelial growth factor (VEFG) in peripheral blood were reduced and tumor necrosis factor increased. Punicalagin, ellagic acid, and gallic acid have an anti‐tumor‐inducing effect. Overall, these findings suggest that pomegranate and its bioactive components could serve as promising adjuncts to conventional prostate cancer therapies, though further investigation is needed to confirm their therapeutic potential (Ma et al. 2015).

### 4.5 Role of Punicalagin in Colon Cancer

Colon cancer has a high mortality rate and is increasing rapidly, making it the third most common kind of cancer worldwide. In the United States last year, an estimated 1.8 million new cases of colorectal cancer were diagnosed. By the year 2030, CRC is expected to lead to an additional 1.1 million deaths and cause another 2.2 million cases globally (Ganesan et al. 2020).

The effect of punicalagin on the expression of Annexin A1 (Anx‐A1) and the interplay between apoptosis and autophagy in HCT 116 colorectal cancer cells was studied by researchers in a recent study. Punicalagin downregulates the Anx‐A1 protein in a colorectal cancer cell line. HCT 116 was used. It modifies protein levels that regulate the measures related to apoptosis and autophagy affected by FPR inhibition. Taken together, these results suggest that these proteins play a part in positively regulating the transcription and translation of punicalagin‐related proteins. Pomegranate peel is the richest source of ellagic acid, another strong bioactive ingredient. Studies have shown that it suppresses cell proliferation as well as inflammation, tumors, and viruses (Kilit and Aydemir 2023a).

The anticancer effects of punicalagin were also studied using three different colon tumor cell lines (HT‐29, LoVo, and HCT 116). Punicalagin was used in a variety of concentrations and treatment times to treat both normal and colon tumor cells. The Cell Counting Kit‐8 (CCK‐8) assay was used to determine the cell survival rate. Researchers used annexin V and a cell death kit to study cell death, and a cell invasion analysis kit to study cell invasion. Proteolytic caspase‐3, matrix metalloproteinase‐2, and matrix metalloproteinase‐9 levels were measured by Western blotting. Early and late apoptosis percentages were added together to determine that punicalagin prompted apoptosis in colorectal tumor cells. Punicalagin therapy generated a substantial increase in caspase‐3 activity. Western blotting experiments verified the increased appearance of stimulated caspase‐3. Punicalagin was shown to inhibit the growth of colon tumor cells. The expression of MMP‐2 and MMP‐9 in colon cancer cells was also suppressed by punicalagin. Inhibiting matrix metalloproteinases‐2 and 9, as well as regulating caspase‐3 activation, was shown to increase the effects of punicalagin on colon cancer cells (Sun et al. 2023).

In another study, the impact of punicalagin (PU) on the interplay between autophagy and apoptosis in HCT 116 colorectal adenocarcinoma cells is analyzed through the regulation of Annexin A1 (Anx‐A1) expression. The inhibitory effects of punicalagin on autophagic, pro‐apoptotic, and Anx‐A1 activities, as determined by selective cytotoxicity, suggest that this polyphenol might possess therapeutic capabilities in colorectal cancer. Using flow cytometry analysis of autophagy flux, it was possible to detect the automatic degradation of autophagosomes in treated cells that were involved in autophagy. The proteomes of 35 unique proteins from punicalagin‐treated cells, both in the presence and absence of Anx‐A1 antagonists, revealed a complex interaction between autophagy and apoptosis. This result implies that punicalagin might, in fact, concurrently stimulate and impede the two cell death mechanisms. Punicalagin has been suggested to have a translational effect by modulating the expression of Anx‐A1 in HCT 116 cells (Ganesan et al. 2020).

Researchers performed extensive screening studies to find natural anticancer compounds, and the most powerful antioxidant components were found in pomegranate peels, that is, granatin B and punicalagin. Researchers studied the effectiveness of these compounds against colon cancer using xenograft tumor models and cultured human HT‐29 colorectal cancer cells. The effects on inflammation and mucositis were studied in rats with Dark Agouti and RAW 264.7 strains that had been generated by lipopolysaccharide, as well as in rats treated with 5‐FU. Pathological coloration, immunofluorescence visualization, western blotting, and flow cytometric studies were used to clarify the underlying processes. Significant anti‐colorectal effects of granatin B and punicalagin were seen in both xenograft and cell culture models of colon cancer. Both granatin B and punicalagin, when administered to 5‐fluorouracil‐treated animals, significantly reduced inflammation and mucositis in RAW264.7 cells that had been stimulated with lipopolysaccharide. Mechanistic analyses showed that punicalagin and granatin B induced apoptosis and S‐phase cell cycle capture in HT‐29 cells via reactive oxygen species. The cell cycle captured in the S phase was also triggered by these chemicals, making HT‐29 cells more vulnerable to the cytotoxicity of 5‐fluorouracil (5‐FU). Future clinical studies should focus on assessing the safety, optimal dosing, and therapeutic efficacy of punicalagin in patients with CRC, particularly in combination with other dietary bioactives or chemotherapeutic agents (Chen et al. 2022).

### 4.6 Punicalagin's Influence on Bladder Cancer

Primary or secondary bone cancer may be referred to as “bone cancer.” A specific kind of primary bone cancer originates from the bone. Chondrosarcoma, ewing sarcoma, and osteosarcoma are all tumors of the primary bone. They comprise less than 1% of annually diagnosed tumors. Osteosarcoma comprises approximately two‐thirds of all cases of bone cancer, making it the most prevalent. Osteosarcoma is identified in approximately 1200 patients annually in the United States. Osteosarcoma progresses from malignant basic mesenchymal cells to osteoblasts, which generate a malignant osteoid matrix upon differentiation. Chondrosarcoma is a kidney‐producing bone malignancy. Its incidence is estimated to be one in 200,000 individuals, making it the least prevalent form of bone cancer (Ferguson and Turner 2018). Pomegranate extracts contain anti‐signaling pathway‐inhibiting polyphenols, which are abundant in these extracts. The peel and mesocarp contain 11–20 g/kg of punicalagin, the most prevalent ellagitannin in pomegranates, while the liquid contains 4–565 mg/L (Rahman et al. 2023). A range of cancer cells, including those with tumors in the bones, breasts, intestines, cervix, and lungs, are susceptible to apoptosis or autophagic cell death, while punicalagin inhibits proliferation (Xie et al. 2022).

Researchers have studied the possibility that punicalagin slowed the growth and invasion of four different types of human cancer cells: U2OS, MG63, SaOS2, and hFOB1.19. The ratio of cancer cell apoptosis was measured using flow cytometry. Western blotting was used to evaluate NF‐κB signaling in these cells. To investigate punicalagin's effects on osteosarcoma progression and angiogenesis in living organisms, the researchers established a skin cancer xenograft model. When osteosarcoma cells were exposed to punicalagin, the cell death rate and proliferation significantly decreased. In a transwell experiment, these cells also considerably reduced the osteosarcoma cells' invasion capacity. Punicalagin caused IκBα degradation and helped p65 escape the nucleus, suggesting that therapy probably inhibited the NFκB signaling pathway. In line with its capacity to decrease NF‐κB activation, punicalagin was shown to substantially reduce levels of interleukin (IL) 6 and IL 8. These effects can be reversed by using inhibitors of NF‐κB. Researchers found that punicalagin exposure decreased in vivo osteosarcoma growth and angiogenesis using a tumor xenograft mice model. The results indicated that Punicalagin prevents osteosarcoma‐related cancers from progressing. The main molecular mechanism that may play a role, perhaps an important one, is blocking the NF‐κB signaling pathway (Huang et al. 2020).

A study was carried out on the antiproliferative and antiangiogenic effects of pomegranate juice (PGJ) in human multiple myeloma cell lines. The study showed that pomegranate juice has antiproliferative properties in multiple myeloma cells and antiangiogenic and G0/G1 cell cycle blocking functionalities. Intriguingly, pomegranate juice was sequentially combined further to boost the cytotoxic action of this proteasome inhibitor. It investigates the effects of pomegranate juice on cell migration and invasion, as well as angiogenesis. Tube formation, microvessel outgrowth, and aortic ring assays were also found to be negatively affected; cell migration and invasion were reduced, as determined by wound‐healing assay or transwell assays. Antiangiogenic properties of pomegranate were validated by the expression evaluation of angiogenic genes in endothelial cells. Thus, treating multiple myeloma with pomegranate juice is an excellent tool for exploring other therapeutic strategies (Tibullo et al. 2016).

Scientists have also conducted another study to determine the anticancer potential of a polysaccharide obtained from pomegranate peels (PP) against human osteosarcoma cancer cells. Pomegranate peels proved to have dose‐dependent influences on U‐2 osteosarcoma (OS) cell growth, arresting the cycle of cells and death. Western blotting analysis shows that by raising the Bax/Bcl‐2 ratio, dropping mitochondrial membrane potentials, and activating caspase‐9 and caspase‐3. Through the mitochondrial intrinsic pathway of cell death, pomegranate peel (PP) inhibits growth in human osteosarcoma tumor cells. However, further mechanistic studies, clinical trials, and improvements in bioavailability are essential for its successful integration into clinical oncology (Li et al. 2014).

### 4.7 Punicalagin's Anticancer Potential in Oral Cancer

Oral cancer contains tumors affecting the lips, as well as every subsite of the mouth and pharynx. Oral cancer is the fifteenth most prevalent cause of mortality globally and the sixteenth most prevalent malignancy, with an age‐adjusted incidence of four cases per 100,000 individuals (Inchingolo et al. 2020). Oral squamous cell carcinomas, which originate from the mucosal epithelium, comprise over 90% of malignant tumors. Nevertheless, it is now recognized that those mucosal carcinomas represent a various diseases. Oral cancer, specifically, should be classified as a distinct illness from carcinoma that originates in the oropharynx. The difference is that oropharyngeal cancer is predominantly related to human papillomavirus (HPV) infection. In contrast, oral cancer is associated with more conventional risk factors such as tobacco and alcohol consumption. Oral squamous cell carcinomas originate in the oral cavity's epithelial lining (Speight and Farthing 2018). The anticancer properties of pomegranate are evaluated through the modulation of signaling pathways in a range of cancer types. The alive cells stopped growing as a result of the punicalagin‐induced change of the signal‐transduction pathway from survival and proliferation to the cell cycle. The development of apoptosis, senescence, and autophagy restricts the progression of cancer (Yasmin et al. 2023).

The potential of pomegranate extract as a natural product to prevent the progression of oral cancer has been studied by researchers. By employing the maceration method and ethanol extraction, pomegranate extract was achieved. This compound decreases ATP synthesis, shortens the sub‐G1 phase, and increases apoptosis, according to the findings. At dosages ranging from 25 to 50 μg/mL, pomegranate extract inhibits the activity of MMP‐2 or MMP‐9 at the microcellular level, thereby inducing pro‐apoptotic, anti‐proliferative, and anti‐angiogenic effects on cancer cells. Pro‐apoptotic and anti‐proliferative effects are produced via mitochondrial injury mechanisms. 72 h exposure is associated with a significant reduction in oral cell viability compared to 24 h exposure. Pomegranate extract potentially affects oral cancer through four separate mechanisms: it inhibits the invasion, migration, and proliferation of oral cancer cells, induces apoptosis in oral cancer cells, and regulates antioxidant genes (Imanu et al. 2023).

Researchers have studied the cytotoxic effects of punicalagin in an in vitro study using a human tongue squamous cell carcinoma cell line. As one of its primary anti‐tumor effects, punicalagin was examined for cytotoxicity using the MMT assay; apoptosis was evaluated using the flow cytometry technique in conjunction with annexin‐v/Propidium iodide; and the ingredient's basic apoptotic action on tongue cancer cells was evaluated. According to the results, the concentration of 185 μmol was half the maximum cytotoxic consequence for Punicalagin. Among the test subjects exposed to 185 μmol/mL of punicalagin, 51.3% had apoptotic cells, 2.3% maintained necrotic cells, and 46.7% possessed cells that were viable. Punicalagin was a reported as an anticancer compound due to its cytotoxic effects on a human tongue squamous cell carcinoma cell line (Sabry et al. 2023).

Researchers have also investigated squamous cell carcinoma cell lines from humans with different formulations of pomegranate extracts, polyethylene glycol, and polylactic acid‐co‐glycolic acid (PE, PEG, and PLGA). Researchers used basic culture conditions to produce and maintain the HNO‐97 human tongue cancer cell line. The cells were separated into two groups according to the culture medium: Polyethylene glycol group (B1) and PE –PEG‐PLGA group (B2). These groups examined cell survival, BAX expression, caspase‐3 expression, and DNA fragmentation. In the first group, diphenylamine was employed, whereas, in the second group, real‐time polymerase chain reaction (RT‐PCR) using (4,5‐dimethylthiazol‐2‐yl)‐2,5‐diphenyltetrazolium bromide was utilized. Cell viability analysis demonstrated that groups B1 and B2 had higher mean values at different concentrations. The control group A showed significantly lower mean values for BAX expression, caspase‐3 expression, and DNA fragmentation than groups B2 and B1. Doses of 6.41 μg/ml and 1.21 μg/ml, respectively, were found to have the same inhibitory concentration (IC50%) in Group B1 and Group B2. Whenever combined with nanoparticles, the pomegranate's anticancer effects are even more prominent (Rageh et al. 2023).

### 4.8 Exploring Punicalagin's Role in Liver Cancer

Liver cancer is the fifth most prevalent cause of cancer‐related mortality in the United States despite being the fifth most prevalent malignancy worldwide. The advanced stage at which liver cancer is frequently detected in patients contributes to the disease's unfavorable prognosis. Over 90% of all cases of liver cancer are hepatocellular carcinomas (HCCs), which are most effectively treated with chemotherapy and immunotherapy. Poor prognosis characterizes liver cancer. 5% to 15% of patients qualify for surgical excision, a procedure that is most appropriate for patients in their early stages (Anwanwan et al. 2020). Numerous cancer cell lines are susceptible to the cytotoxic and growth‐suppressing effects of pomegranate fruit extract. Punicalagin (2,3‐hexahydroxydiphenylgallagil‐D‐glucose), which is present in significant amounts in *P. granatum* , is a bioactive tannin compound of pomegranate peel. It possesses a high degree of bioavailability and is the largest water‐soluble ellagitannin molecule (Alqobi et al. 2016).

Researchers have synthesized punicalagin nanoparticles and then tested their antioxidant, antibacterial, and antiproliferative effects on HepG2 cancer cells. The average size of punicalagin nanoparticles was found to be 87 nm by the use of dynamic light scattering. However, scanning electron microscopy analysis demonstrated the existence of spherical nanoparticles ranging in size from 90 to 116 nm. The presence of punicalagin in produced nanoparticles was verified by HPLC‐based research. Additionally, the findings indicated that punicalagin nanoparticles exhibited antioxidant activity nearly four times that of the bulk and had an inhibition zone measuring approximately 13 mm. According to the results of the antiproliferative assay, the nanoparticles of punicalagin inhibited the viability of malignant cells by nearly 44% at 100 μg/mL. In contrast, the bulk form exhibited a mere 15% reduction. In contrast to its mass counterparts, the study indicates that the herbal substance in nano form has a substantial therapeutic potential for cancer (Mehra et al. 2021).

In another study, researchers studied the potential for polyphenol‐rich, naturally occurring products to halt the progression of HCC. Diethylnitrosamine (DENA) induces hepatocarcinogenesis in male albino rats, and the study observed the chemopreventive effects of punicalagin and pomegranate juice (PJ) against this disease. The animals were randomly assigned to one of six groups for 11 weeks and subjected to varying treatments. Group 1 served as a control; Group 2 received 10 mL of pomegranate juice (PJ) per kg body weight (bw) orally, group 3 received 18.5 mg PCG/kg body weight (bw), and groups 4–6 were given an intraperitoneal dose of DENA (50 mg/kg bw) once a week. The hepatocellular carcinoma (HCC) group (treated with DENA) made up Group 4, whereas HCC + PJ and PCG were in Groups 5 and 6, respectively. Pomegranate juice inhibited DENA‐induced increases in NO, GST, MDA, TNF‐α, NF‐κB‐p65, ALAT, and TNF‐α and restored levels of total protein, IL‐10, SOD, and CAT, according to the findings. Additionally, |Pomegranate juice PJ led to the downregulation of the mRNA expressions of Bcl‐2, Bcl‐21, and Bcl‐XL, while it increased the expressions of caspase‐3 and Bax. PJ's chemopreventive actions similarly reduced the hepatic preneoplastic lesions generated by DENA. While punicalagin treatment produced some modulation in rodents treated with DENA, it failed to demonstrate significant chemopreventive activity and instead caused certain side effects. Apoptosis was induced, and miR‐21 expression was downregulated by both punicalagin and pomegranate juice (Hussein et al. 2022).

**FIGURE 3:**
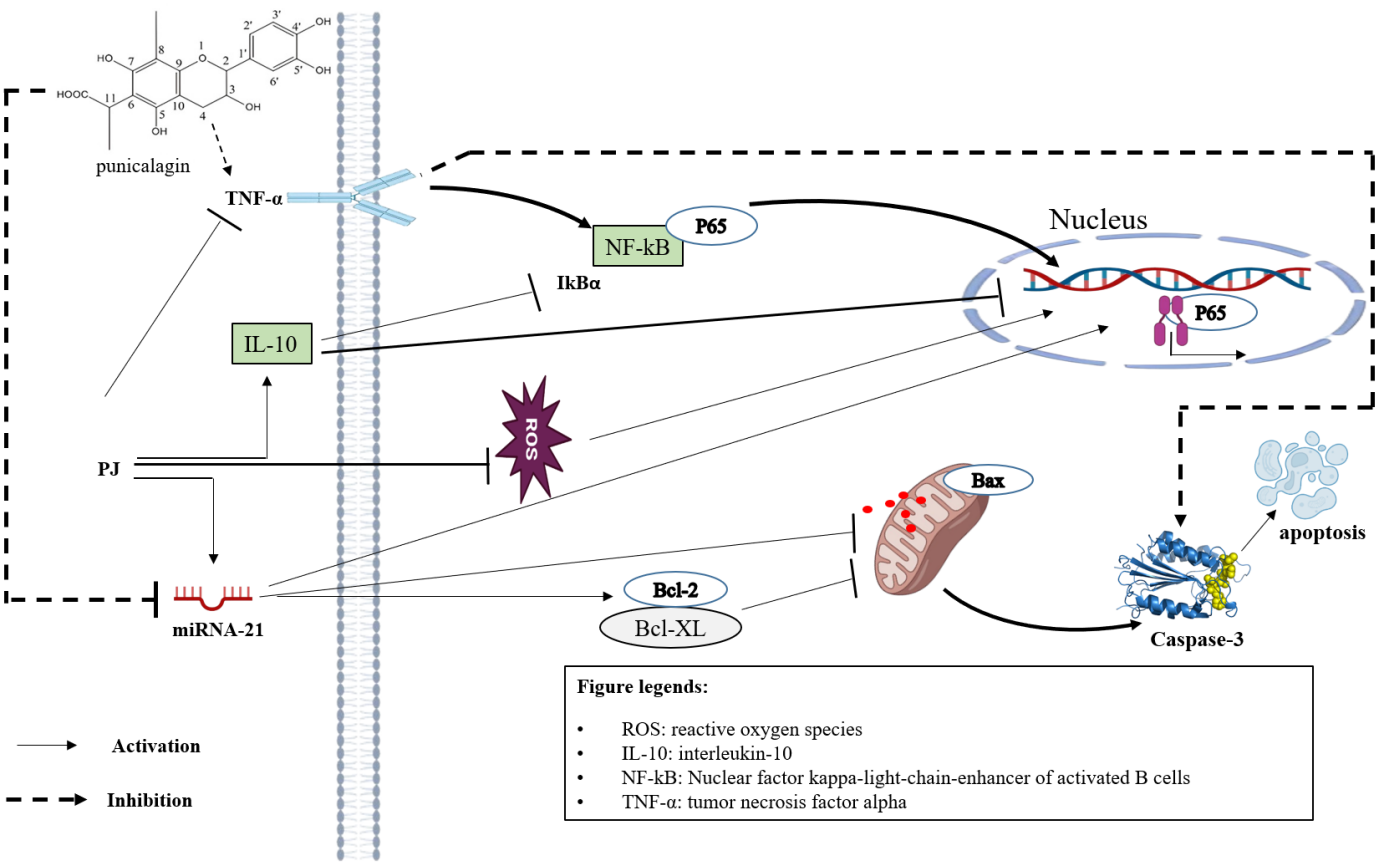
Molecular mechanism involved in chemoprevention of hepatocellular cancer mediated by pomegranate juice (PJ) and punicalagin (PCG). A schematic representation detailing the molecular interactions through which pomegranate juice and punicalagin exert chemopreventive effects against hepatocellular carcinoma (HCC). The figure includes the activation of antioxidant defense, modulation of inflammatory pathways, and the inhibition of carcinogenic signaling cascades, emphasizing key players such as Nrf2, STAT3, and ROS balance.

To determine the potential mechanisms of punicalagin and ellagic acid, the researchers studied their distinct anticancer effects on HepG2 cells. Cell division, cell shape, cell cycle progression, and programmed cell death were examined. Cell‐cycle arrest in the S‐phase and G0/G1‐phase was mediated by punicalagin and ellagic acid, resulting in apoptosis in HepG2 cells. Furthermore, an increase in intracellular H_2_O_2_ production and the activation of apoptosis‐related protein activities were simultaneously observed in the levels of reactive oxygen species. Both punicalagin and ellagic acid were shown to cause apoptosis in HepG2 cells. However, punicalagin was more efficient in suppressing cell growth. Future research could explore the synergy between punicalagin and chemotherapy, particularly in xenograft tumor models, to better understand its role in combination therapies (Li et al. 2019). The molecular mechanism involved in the chemoprevention of hepatocellular cancer mediated by pomegranate juice (PJ) and punicalagin (PCG) is presented in Figure 3.

### 4.9 Punicalagin in Gastric Ulcer Treatment

Gastric ulcers are the most prevalent disease affecting the upper digestive tract. Gastric ulcers occur with an annual incidence rate of 0.10%–0.19% in Western populations, making them 2.4% common. In some parts of Mainland China, the prevalence of gastric ulcers can reach 22.5% among patients and 6.07% among the general population. Gastric ulcers are characterized by gastrointestinal symptoms (Bi et al. 2014). A substantial breach in the integrity of the mucosal barrier distinguishes one prevalent chronic gastrointestinal disorder, gastric ulcer. Prominent determinants contributing to the development of gastric ulcers are the frequent administration of non‐steroidal anti‐inflammatory drugs and *Helicobacter pylori* ( *H. pylori* ) infection. Antibiotics and acid inhibitors are frequently employed in the treatment of gastric ulcers (Khoder et al. 2016). Punicalagin (C48H28O30), an ellagitannin primarily present in pomegranate skin, is a highly potent antioxidant found in pomegranate juice. It has been established that pomegranate juice possesses properties that include anticancer, anti‐inflammatory, antiviral, antiproliferative, and antioxidant effects (Rakshit et al. 2020). When used to block recurring disease‐causing viruses (HSV, RSV, and HSV‐1), punicalagin showed prospects as a broad‐spectrum antiviral drug. Through interactions between viral glycoproteins and glycosaminoglycans on cell surfaces, these viruses are able to enter host cells (Venusova et al. 2021).

Research on the antiulcerogenic effects of a methanol extract from pomegranate peel was conducted using male Wistar albino rats. The gastric mucosa was protected against the negative effects of indomethacin (50 mg/kg) through a 15‐day oral pretreatment with peel extracts (25, 50, and 100 mg/kg). 100% of the control cohort exhibited ulceration. In comparison to the group that developed gastric ulcers induced by indomethacin, the most favorable outcomes were observed in the acidic summer cultivar at a dosage of 50 mg/kg, where peptic ulcer inhibition was observed. The ulcer index was found to be the lowest (5.4 ± 0.55), and there was a significant decrease in the migration of polymorphonuclear leukocytes and blood loss when sour summer extracts (50 mg/kg) were given. Research suggests that pomegranate peel extract may have healing properties as an antiulcer, especially in the summer, and its high antioxidant activity might cause this. The potential financial benefits and positive nutritional attributes of this product may be attributed to the outcomes derived from pomegranate peel extract (Ghazaleh et al. 2013).

In an interesting study, researchers studied punicalagin's potential gastroprotective effects against stomach damage caused by ethanol. As a reference drug, rats in Group 1 received 5 mL/kg of absolute ethanol through the abdomen; rats in Group 2 received ethanol alone; rats in Group 3 were pre‐treated with ranitidine at 50 mg/kg via the abdomen; and rats in Group 4 were pre‐treated with punicalagin at 4 mg/kg injecting fluid. In addition to reducing ethanol‐induced oxidative stress, ulcer index, and histological changes, pretreatment with PCG enhanced antioxidant activity. The concentration of IL‐10 and other inflammatory cytokines, such as TNF‐α, IL‐1β, and IFNγ, was decreased. At the same time, the expression of the Tumor Necrosis Factor (TNF‐α) gene was increased in the mucosa. It also decreased myeloperoxidase and caspase 3 activity, caspase 9 gene expression, and NF‐κB protein expression in the mucosa. However, it did increase mucin content, antiapoptotic B‐Cell Lymphoma 2 (Bcl‐2) gene expression, and mucosal nitric oxide generation. The results revealed more evidence that punicalagin can protect the stomach against ethanol‐induced ulcers. The outcome was a reduction in inflammation and mucosal oxidative stress through the NF‐κB pathway. The cytoprotective mechanisms provided by punicalagin are independent of prostaglandin E2 and acid secretions (Katary and Salahuddin 2017).

Using rats on a diet supplemented with pomegranate peel powder, scientists examined the efficacy of an alternative treatment for ulcer prevention, particularly in relation to aspirin. Group (1) served as the control, Group II was an ulcer group, and Group III was the ulcer‐supplemented group. A total of 21 male rodents weighing between 140 and 170 g were utilized for the biological evaluation. Four weeks were given for the evaluation. Suspensions of aspirin in water (500 mg/kg rodent weight) induced gastric ulceration. The COX‐2 and TNF‐α gene expression levels were determined using real‐time polymerase chain reaction (RT‐PCR). Gastric ulcer area and ulcer index, gastric fluid volume, and acidity were all decreased by pomegranate peel powder (10% w/w), according to the findings. Histological examination showed that pomegranate powder restores gastric mucus and gastric tissue. By supplementing plasma with pomegranate peel powder, nitric oxide production increased while TNF‐α levels decreased. The pomegranate peel powder resulted in a significant downregulation of the expression of the TNF‐α and COX‐2 genes in the gastric mucosa (changes of 2.4 and 12.5 fold, respectively). A protective dietary supplement against gastric ulcers is pomegranate peel powder (10%) (Mohamed and Mabrok 2021). Renal cancer comprises renal cell carcinoma (RCC), which is the most prevalent variety, and renal transitional cell carcinoma (RTCC), which develops in the renal pelvis and parenchyma, respectively. Primarily for early‐stage cancers, the incidence of RCC has increased in the United States (Chow et al. 2010). Kidney cancer ranks among the 10 most prevalent forms of cancer in Western societies. An estimated 270,000 cases of renal cancer are identified annually on a global scale, resulting in 116,000 mortalities. Renal cell carcinomas (RCCs) comprise an estimated 90% of all kidney tumors (Ljungberg et al. 2011). Surgery can cure RCC, although a small percentage of patients may experience a recurrence. Natural remedies exist for these tumors, one of which is punicalagin (PN), a phenolic compound discovered in pomegranate juice. It has anti‐cancer and antioxidant properties (Aladaileh et al. 2021). In addition to inhibiting angiogenesis, invasion, and proliferation, punicalagin treatment has been demonstrated to cause autophagic and apoptotic cell death in numerous types of cancer (Kilit and Aydemir 2023b).

Researchers have studied the biochemical impacts of punicalagin extract (PE), or pomegranate juice, on renal cell carcinoma (RCC) cell lines. The effects of PE on NF‐κB, JNK, and the EMT phenotype in pVHL‐deficient renal cell carcinoma RCCs were studied in a preclinical condition. This included whether punicalagin extract affected invasion, anchorage‐independent growth, and proliferation. The results showed that punicalagin extract blocks the EMT phenotype of pVHL‐deficient ccRCCs by inhibiting the NF‐κB and JNK signaling pathways. Concentration determines the impact of punicalagin extract on biochemical indicators of EMT, such as cadherin expression, in addition to functional manifestations of EMT, such as invasion. Within days, these effects become noticeable when PE is diluted a thousandfold. It was found that after long‐term exposure, very low concentrations of PE (106 dilution) may inhibit NF‐κB and JNK and reverse the EMT phenotype. It is worth considering PE as an additional dietary supplement to active surveillance for tiny, locally located, incidentally found kidney tumors. This might help in patient care and avoid the need for nephrectomy and other invasive treatments. Future studies could focus on how punicalagin interacts with other apoptosis‐related proteins and whether combining it with other cancer therapies could enhance its efficacy (An et al. 2015).

### 4.10 Effect of Punicalagin in Blood Cancer

Blood cancer is a disease characterized by the destruction of red blood cells. In 2019, the mortality toll attributed to this ailment amounts to around 1500 individuals, representing less than 0.2% of the overall impact of blood cancer exclusively. Each year, approximately 20,000 individuals in the United States receive a diagnosis of myeloma. Lymphoma, leukemia, and myeloma are the three primary forms of blood tumors. Acute Lymphocytic Leukemia (ALL) is a type of white blood cell tumor in which the bone marrow becomes infected. “Acute” denotes the disease's rapid progression; therefore, failure to receive treatment during its early stages could potentially result in mortality within a short period. The classifications for ALL are L1, L2, and L3. Plasma, an embryonic teratoma of cells that assists in the removal of the infection, is present in multiple myeloma (MM) (Kumar et al. 2020). Punicalagin (C48H28O30), an ellagitannin found primarily in pomegranate skin and pomegranate juice, is a potent antioxidant with anticancer properties (Rakshit et al. 2020). There are numerous health benefits associated with pomegranate juice, including anti‐inflammatory, cancer‐preventative, and inhibitory effects (Chen et al. 2023). Pomegranate's anticancer properties involve the regulation of signaling pathways in a range of tumors, including blood cancer, prostate cancer, and breast cancer. By halting living cells, punicalagin induces an alteration of the signal‐transduction pathway away from survival and proliferation and toward the cell cycle. Apoptosis, senescence, and autophagy inhibit the progression of cancer (Yasmin et al. 2023).

Utilizing NB4 and MOLT‐4 leukemic cell lines, researchers examine the molecular mechanism and anti‐leukemic effects of punicalagin. Before evaluating cell viability with an MTS experiment, leukemic cells were treated with punicalagin. Flow cytometry was employed to measure apoptosis and autophagy, respectively, with anti‐LC3/FITC and Annexin V‐FITC/PI antibodies. Utilizing reverse transcription‐quantitative PCR, apoptotic and autophagic mRNA levels were established. According to the findings, the viability of NB4 and MOLT‐4 cells was found to be dose‐dependently decreased by punicalagin. A synergistic cytotoxic effect was observed when punicalagin was combined with daunorubicin; by upregulating the expression of Bax and caspase‐3/−8/−9, punicalagin induced apoptosis. Bcl‐2 expression was consequently decreased. By inhibiting mTOR and increasing ULK1 expression, punicalagin additionally facilitated autophagy. By regulating autophagy via the mTOR/ULK1 signaling pathway, activating the caspase cascade, and modulating Bax and Bcl‐2, punicalagin decreases cell proliferation and enhances cell death and autophagy (Subkorn et al. 2021).

The antiproliferative and apoptotic effects of polyphenolic‐rich extracts derived from the non‐edible portions of *P. granatum* on U266 multiple myeloma cells were investigated. The study provides evidence that the proliferation of U266 cells decreased in a dose‐dependent manner when exposed to extracts of *P. granatum* . Furthermore, U266 cells that were exposed to the stem and leaf extracts experienced apoptosis and a substantial increase in mitochondrial membrane potential (MMP) loss; the flower extract caused only a marginal increase in MMP loss. The results showed that *P. granatum* extracts caused cytotoxicity and cell death in U266 multiple myeloma cells from humans. This was accomplished through the inhibition of mitochondrial membrane potential and an increase in cell cycle arrest. The evidence indicates that the extracts may have potential applications in cancer chemoprevention (Kiraz et al. 2016).

Researchers have aimed to investigate pomegranate juice's (PGJ) antiproliferative and antiangiogenic characteristics in mouse models of multiple myeloma. Multiple myeloma (MM) cells exhibit anti‐angiogenic, anti‐proliferative, and G0/G1 cell cycle inhibitory effects in response to pomegranate juice. The study investigated the effects of pomegranate juice on angiogenesis and cell migration/invasion using wound healing and transwell assays, respectively. Results showed that pomegranate juice inhibited cell migration and invasion, as well as micro‐vessel outgrowth, sorting ring, and tube formation. Endothelial cell expression of angiogenic genes was influenced by pomegranate, demonstrating its anti‐angiogenic abilities. As a result, the pomegranate juice administration could help as a valuable component for the progress of novel therapeutic methods to treat multiple myeloma (MM) by capitalizing on its anti‐angiogenic and anti‐proliferative properties (Tibullo et al. 2016).

### 4.11 Targeting Brain Cancer With Punicalagin

Brain tumors are pathologies characterized by abnormal cell growth and division in brain tissue. Primary and metastatic brain tumors are terms used to indicate the two main types of brain tumors, which differ in their origin. A sort of brain tumor recognized as a glioma develops from glial cells (Işın et al. 2016). The pomegranate peel contains punicalagin, a bioactive medicinal component with a high molecular weight that is similarly prevalent in the fruit's seeds and juice. The anti‐oxidant and anti‐tumor properties of punicalagin have been demonstrated (Kilit and Aydemir 2023b). It has been found that it inhibits tumor cell growth and has anti‐proliferative and apoptotic properties. Reducing cell viability and increasing cyclin E, punicalagin produced from pomegranates (1–30 μg/mL) when administered for 24 h showed promising results. Further evidence of autophagy was shown in U87MG cells in the form of a punctate pattern labeled with a green fluorescence‐LC3 fusion protein (GFP‐LC3‐II), which was enhanced by the treatment. To induce autophagic cell death, punicalagin elevated AMPK and p27 phosphorylation levels (Wong et al. 2021).

In another study, the in vitro effects of punicalagin on U87MG glioma cells derived from humans were observed. Using the MTT test, researchers determined if the U87MG glioma cells from humans were viable. In order to identify the cell cycle, flow cytometry was employed. Immunoblot analysis was used to evaluate the amounts of phosphor‐AMPK, cleaved poly (ADP‐ribose) polymerase (PARP), cleaved caspase‐9, Bcl‐2, and phosphor‐p27 at Thr198. A spectrophotometer was used to quantify the caspase‐3 activity. Analysis of LC3 cleavage and punctate patterns was performed to ascertain autophagy. The results showed that punicalagin (1–30 μg/mL) influenced cell survival in a dose‐dependent way, along with increased cyclin E levels and decreased cyclin B and cyclin A levels. The therapy also caused cell death by activating apoptosis markers such as PARP cleavage, caspase‐9 activation, and caspase‐3 activity rise. Despite treating the cells with the pan‐caspase inhibitor z‐DEVD‐fmk (50 μmol/L) previously, cell death was still partially prevented. Punicalagin treatment, in contrast, enhanced LC3‐II cleavage and resulted in a punctate pattern in the cells labeled with GFP‐LC3‐II. A dose‐dependent reduction in cell death induced by punicalagin was seen when chloroquine (1–10 μmol/L) was administered to inhibit cell autophagy. Punicalagin (1–30 μg/mL) caused an increase in phosphor‐AMPK and phosphor‐p27 at Thr198 in the cells, which led to the activation of autophagic cell death. In human U87MG glioma cells, punicalagin causes cell death by apoptosis and autophagy (Wang et al. 2013).

Another research study was conducted to determine the anti‐glioma effects of punicalagin extract nano‐emulsions (NEs) on C6 cells by measuring their cytotoxicity. Utilizing punicalagin extract concentrations of 1.5 and 3.0%, NEs were synthesized by the spontaneous emulsification process. Subsequently, their antioxidant activity and physical stability were tested. During a 72‐h incubation period, markers of toxicity such as genotoxicity, cell survival, catalase activity, protein carbonylation, lipid peroxidation, and hemolysis were evaluated in human blood cells at doses of 0.1, 0.25, and 0.5 mg/mL punicalagin extract. Testing the anticancer effects on glioma cells in vitro at 24 and 48 h was done using astrocytes, a non‐transformed cell type, as a control. Punicalagin extract, which has a detoxification power of around 30%, increased cell survival by 50% by stimulating the proliferation of mononuclear cells. The results of the tests for catalase activity, protein carbonylation, and lipid peroxidation did not indicate that NEs had any oxidative or genotoxic damage. High amounts had a hemolytic impact, according to the hemolysis investigation. On top of that, formulations decreased tumor cell viability to about 47%. Finally, nanoemulsions showed potential as a therapy for brain tumors based on their in vitro anticancer activity. However, further research is required to fully understand their mechanism of action, optimize delivery methods, and assess their clinical effectiveness in human trials (Mota Ferreira et al. 2016).

### 4.12 Punicalagin's Therapeutic Role in Cervical Cancer

Researchers studied the in vitro effects of punicalagin on U87MG glioma cells derived from humans. Using the MTT test, researchers determined if the U87MG glioma cells from humans were viable. In order to identify the cell cycle, flow cytometry was employed. Immunoblot analysis was used to evaluate the amounts of phosphor‐AMPK, cleaved poly (ADP‐ribose) polymerase (PARP), cleaved caspase‐9, Bcl‐2, and phosphor‐p27 at Thr198. A spectrophotometer was used to quantify the caspase‐3 activity. Analysis of LC3 cleavage and punctate patterns was performed to ascertain autophagy. The results showed that punicalagin (1–30 μg/mL) influenced cell survival in a dose‐dependent way, along with increased cyclin E levels and decreased cyclin B and cyclin A levels. The therapy also caused cell death by activating apoptosis markers such as PARP cleavage, caspase‐9 activation, and caspase‐3 activity rise. Despite treating the cells with the pan‐caspase inhibitor z‐DEVD‐fmk (50 μmol/L) previously, cell death was still partially prevented. Punicalagin treatment, in contrast, enhanced LC3‐II cleavage and resulted in a punctate pattern in the cells labeled with GFP‐LC3‐II. A dose‐dependent reduction in cell death induced by punicalagin was seen when chloroquine (1–10 μmol/L) was administered to inhibit cell autophagy. Punicalagin (1–30 μg/mL) caused an increase in phosphor‐AMPK and phosphor‐p27 at Thr198 in the cells, which led to the activation of autophagic cell death. In human U87MG glioma cells, punicalagin causes cell death by apoptosis and autophagy (Wang et al. 2013). To analyze the anti‐invasive and anti‐migration effects of pomegranate on U87 cells, researchers utilized the 3D collagen invasion test and the 2‐scratch assay. The results demonstrated that in a way that was both dose‐ and time‐dependent, pomegranate juice decreased the invasion of U87‐MG spheroids through collagen. Moreover, in the scratch experiment, pomegranate juice inhibited the migration of U87‐MG cells more effectively in dosage and duration. A study found that analyzing pomegranate juice's influence on glioma cell invasion was more complex using the 3D model (Zraikat et al. 2018).

Another research study was conducted to study the anti‐glioma effects of punicalagin extract nano‐emulsions (NEs) on C6 cells by measuring their cytotoxicity. Utilizing punicalagin extract concentrations of 1.5% and 3.0%, NEs were synthesized by the spontaneous emulsification process. Subsequently, their antioxidant activity and physical stability were tested. During a 72‐h incubation period, markers of toxicity such as genotoxicity, cell survival, catalase activity, protein carbonylation, lipid peroxidation, and hemolysis were evaluated in human blood cells at doses of 0.1, 0.25, and 0.5 mg/mL punicalagin extract. The anticancer effects on glioma cells in vitro were tested at 24 and 48 h using astrocytes, a non‐transformed cell type, as a control. Punicalagin extract, which has a detoxification power of around 30%, increased cell survival by 50% by stimulating the proliferation of mononuclear cells. The results of the tests for catalase activity, protein carbonylation, and lipid peroxidation did not indicate that NEs had any oxidative or genotoxic damage. High amounts had a hemolytic impact, according to the hemolysis investigation. On top of that, formulations decreased tumor cell viability to about 47%. Finally, nanoemulsions showed potential as a therapy for brain tumors based on their in vitro anticancer activity (Mota Ferreira et al. 2016).

### 4.13 Combating Thyroid Cancer With Punicalagin

Thyroid cancer constitutes 1% of all cancers worldwide and is the most prevalent endocrine malignancy in humans. Over the past half‐century, there has been a substantial surge in the prevalence of thyroid cancer. There are thyroid malignancies that develop from parafollicular cells (C cells) and follicular cells. Differentiated thyroid cancer encompasses a range of malignancies that arise from follicular cells; these include anaplastic carcinoma, poorly differentiated carcinoma, papillary carcinoma, and follicular carcinoma. Thyroid cancer has been progressively becoming more prevalent, with an estimated 53,990 cases diagnosed in the United States of America by 2018. Approximately 3% of all malignant tumors in humans are classified as this neoplasm, which is the most prevalent endocrine tumor. Among individuals under the age of 55, two‐thirds of cases occur, and 75% of cases occur in women (Arrangoiz et al. 2019). At present, over 60% of anticancer compounds that have been found beneficial for individuals with cancer are derived from microbiological and medicinal sources (Sharifi‐Rad et al. 2020). There are several bioactive components in punicalagin (PN), some of which include antioxidant, anti‐inflammatory, anti‐viral, antiproliferative, and anticancer effects. A multitude of studies have demonstrated the therapeutic effects of punicalagin, including the inhibition of proliferation, invasion, and angiogenesis, as well as the activation of programmed cell death pathways like apoptosis and autophagy in many cancers, including papillary thyroid, ovarian, and breast (Kilit and Aydemir 2023b). Researchers reported that punicalagin inhibited the viability of the BCPAP thyroid cancer cell line. The distribution of the cell cycle and nuclear fragmentation, chromatin condensation, and BCPAP cells were not impacted by punicalagin treatment. Furthermore, the destruction of caspase‐3 and PARP was not detected due to punicalagin treatment. These attributes collectively demonstrated that punicalagin‐induced BCPAP cells undergo non‐apoptotic cell demise. Cancer cells initiate autophagy in response to a variety of anticancer therapies. The treatment of cells with punicalagin resulted in autophagy induction, as shown by an enhancement of LC3‐II conversion, beclin‐1 expression, and p62 degradation. In addition, the autophagy inhibitor 3‐methyladenine was discovered to limit the stimulation of cell death by punicalagin significantly. Additionally, punicalagin suppresses the mTOR signaling pathways and stimulates the MAPK in an attempt to promote autophagy. The collective findings offer support for the concept that punicalagin possesses an antitumor effect, which is significantly associated with its capacity to stimulate autophagic cell mortality (Cheng et al. 2016).

Researchers studied punicalagin's potential to stimulate autophagic cell death in papillary thyroid cancer cells. As DNA damage may be shown to induce autophagy, PUN causes DNA damage, which is associated with cell death. Increased phosphorylation of histone H2A.X showed that DNA breaks occurred after punicalagin therapy. The DNA damage caused by punicalagin did not include the accused reactive oxygen species or a change in DNA structure. When compared to ataxia telangiectasia and Rad3‐related protein (ATR), phosphorylation of ataxia telangiectasia mutant gene‐encoded protein (ATM) was elevated in a time‐ and dosage‐dependent manner following punicalagin therapy. The ATM inhibitor KU‐55933 blocked punicalagin‐induced ATM phosphorylation, and the resulting decrease in cellular viability was restored. In papillary thyroid cancer cells, punicalagin has been demonstrated to trigger cell death by activating an ATM‐mediated DNA damage response, thereby revealing potential targets and novel mechanisms that contribute to a greater comprehension of the anticancer effects of punicalagin (Yao et al. 2017).

Punicalagin administration caused a senescent phenotype in BCPAP cells, as assessed by senescence‐associated β‐galactosidase (SA‐β‐Gal) staining and altered shape, as reported in a research study. In addition to p21 protein upregulation and cell cycle arrest, punicalagin administration was found to promote senescence. On the other hand, the senescence‐associated secretory phenotype (SASP) was characterized by elevated concentrations of pro‐inflammatory cytokines, specifically IL‐6 and IL‐1β. Punicalagin exposure was also associated with p65 nuclear translocation and subsequent phosphorylation and degradation of IκBα. These findings indicate that the NF‐κB signaling pathway was activated. The specific activator of NF‐κB pyrrolidine dithiocarbamate (PDTC) partially restored the cell death phenotype caused by punicalagin in BCPAP cells, as demonstrated by the reduction in the proportion of SA‐β‐Gal‐positive cells and the suppression of SASP production. The results show that treatment with punicalagin activates nuclear factor‐kappa B, which in turn causes senescent growth arrest and SASP. Further research into its impact on other cellular components and pathways could refine its therapeutic application in cervical cancer treatment (Cheng et al. 2018).

### 4.14 Punicalagin's Potential Against Lung Cancer

Lung cancer is a primary contributor to both the development and mortality of cancer worldwide, with an approximate record of 2 million diagnoses and 1.8 million deaths. Lung neoplasms rank as the second most prevalent form of cancer among both males and females. The incidence of lung cancer is on the rise worldwide due to the industrialization of developing nations and the increased availability of tobacco (Thandra et al. 2021). Recent studies have shown that pomegranate has therapeutic properties through modulating signaling pathways in different forms of cancer (Yasmin et al. 2023).

The cytotoxic effects of punicalagin at concentrations of 50 μM and 75 μM on the lung tumor cell line A549 and the lung epithelial cell line MRC‐5 were studied in recent research, and it was determined that punicalagin is an effective promoter of apoptosis. Although punicalagin exhibits cytotoxic effects on the A549 cell line, its inability to induce a toxic response in the epithelial cell line suggests that it possesses selective toxicity. This characteristic is critical in the development of cancer therapeutics. Furthermore, reports indicate a reduction in cytoplasmic reactive oxygen species (ROS) production as well as an increase in the release of superoxide radicals from mitochondria. These changes in cellular morphology and the activation of caspases to break down PARP provide further evidence in favor of the induction of the apoptosis pathway (Kilit and Aydemir 2023b).

The potential of punicalagin to inhibit the proliferation of lung cancer A549 cells was determined by inducing cell death and blocking STAT‐3 activation. Punicalagin has a dose‐dependent harmful effect on A549 cells after 24 h of treatment. Punicalagin (10, 20, and 30 μM) also caused A549 cells to produce reactive oxygen species, change their mitochondrial membrane potential, and exhibit morphological abnormalities linked to cell death. Overexpression of STAT‐3 regulates apoptosis, proliferation, and angiogenesis. Punicalagin blocked STAT‐3 translocation and caused apoptosis in A549 cells by reducing Bcl‐2 expression and elevating Bax, cytochrome‐c, caspase‐9, and caspase‐3. Consequently, punicalagin is proposed as a potential therapeutic intervention for non‐small cell lung cancers (Fang et al. 2021).

In an interesting study, MRC‐5 and A549 cell lines were used to study punicalagin's antiproliferative mechanism in normal lung fibroblast cells and adenocarcinoma individual alveolar basal epithelial cells, respectively. For 24 h, cultured cells were injected with punicalagin at doses ranging from 1 to 100 μM. Inhibition of cell growth, proportion of apoptotic cells, distribution of the cell cycle, morphological alterations, production of reactive oxygen species (ROS) by cells, mitochondria, or expression of apoptotic proteins were examined. Cell viability and morphological analyses found that punicalagin exhibited a toxic effect against lung cancer cells but not normal lung cells at concentrations of 50 and 75 μM. With the application of punicalagin, cytoplasmic ROS production decreased. In contrast, mitochondrial dysfunction increased the level of ROS released. Research on apoptosis revealed that A549 cells were induced to undergo apoptosis by both concentrations of punicalagin. The cell cycle, nevertheless, stopped in the G (1)/S phase after treatment with punicalagin. The results showed that at these concentrations, punicalagin exhibits antiproliferative and apoptotic characteristics (Berköz and Krosniak 2020).

To test the therapeutic effects of (pomegranate) leaf extract, animal lung cancer cells (A549/H1299) and human lung cancer (NSCLC) were used in an experiment. Researchers also investigated the fundamental mechanisms of action of pomegranate leaf extract (PLE; rich in punicalagin). Findings demonstrate that pomegranate leaf extract concentration and time‐dependently inhibited cell proliferation in a non‐small‐cell lung cancer cell line. Experiment results from flow cytometry (FCM) showed that pomegranate leaf extract (PLE) affected H1299 cell viability by dose‐dependent apoptosis induction and G2/M phase cell cycle progression inhibition. In addition to lowering mitochondrial membrane potential and reactive oxygen species (ROS), pomegranate leaf extract may trigger apoptosis through the mitochondria‐mediated apoptotic pathway. In vitro, pomegranate leaf extract (PLE) inhibited the migration and invasion of H1299 cells and lowered levels of matrix metalloproteinases (MMPs) MMP‐2 and MMP‐9. Based on the results, pomegranate leaf extract (PLE) showed promise as a non‐toxic and effective chemotherapeutic drug for NSCLC therapy. It can prevent cell migration and invasion, induce apoptosis, and reduce growth (Li et al. 2016).

### 4.15 Punicalagin's Impact on Bladder Cancer

Bladder cancer (BC) is the most prominent gastrointestinal cancer worldwide. BC has been identified as the eighth most prevalent form of cancer among women, whereas it ranks fourth among males on the list of the most prevalent cancers. Annually, over 550,000 new cases of bladder cancer (BC) and 200,000 BC‐related injuries are reported globally. The regions analyzed exhibited the greatest incidence rates, including those in North America and southern and western Europe (Wigner et al. 2022). Due to the increased rates of progression and recurrence, radical cystectomy and neoadjuvant chemotherapy are the standard treatments for bladder cancer that has expanded to the muscle layer (DeGeorge et al. 2017). Punicalagin (C48H28O30), an ellagitannin found primarily in pomegranate peel and an active antioxidant in pomegranate juice, is among the numerous polyphenols and antioxidants that inhibit bladder cancer (Rakshit et al. 2020). To trigger apoptosis in tumor cells, PEPE2 treatment likely disrupted the HSP90/Akt‐1/Ask‐1 pathway, as evidenced by the upregulation of ser83 phosphorylation and the upregulation of Thr845 phosphorylation of ASK‐1 in UBUC cells. The negative regulation of the JAK/STAT3 pathway, which controls cell propagation, has been documented in numerous observations (Huang et al. 2022).

In another study, 80 Sprague Dawley male rats were separated into four groups to determine if pomegranate juice (PJ; rich in punicalagin) can prevent bladder cancer. Urinary bladder tissues of all rats were examined histopathologically and immunohistochemically (p53) for oxidative stress marker analysis, tumor protein p53 (TP53), and the expression of interleukin 6 (IL‐6), tumor necrosis factor‐alpha (TNF‐α), and hypoxia‐inducible factor 1 (HIF‐1) were evaluated after their sacrifice after 12 weeks. In the groups that were not affected by cancer, PJ, and normal, the incidence of bladder cancer (BC) was 0%; however, in the group that had induced cancer, the incidence was 20% (or 100%). Cancer‐induced group p53 immunostaining was significantly elevated, whereas cancer‐prohibited group levels were considerably lower. In the cancer‐prevented cohort, a reduction in oxidative stress markers was detected. The unexpected inhibitory impact of pomegranate juice (PJ) on the formation of bladder cancer (BC) is likely due to its antibacterial and antioxidant characteristics (Mortada et al. 2020).

Researchers have also studied the molecular mechanism by which pomegranate juice (rich in punicalagin) prevents urothelial carcinoma of the urinary bladder. Using tandem mass spectrometry and two‐dimensional gel electrophoresis, researchers identified the deregulated proteins. Comparative proteomics results showed that ethanol extract differently expressed 20 proteins in T24 cells, with one protein being down‐regulated and 19 proteins being up‐regulated. Such amorphous proteins affected aerobic glycolysis, cell proliferation, proteasome activity, cytoskeleton control, and apoptosis. An additional study on signaling pathways has shown that treatment with ethanol extract may limit the growth of urinary bladder urothelial carcinoma cells through suppression of the PTEN/AKT/mTORC1 pathway via profilin overexpression (Wu et al. 2016).

A research study was conducted to determine the exact molecular pathway by which the naturally occurring pomegranate fruit can decrease urothelial carcinoma growth in the urinary bladder. The experiment with 3‐(4,5‐dimethylthiazol‐2‐yl)‐2,5‐diphenyltetrazolium bromide showed that the pomegranate pulp extract had less inhibitory action than the ethanol extract from the peel when it came to human urinary bladder urothelial carcinoma cells (UBUC) T24 and J82. Further research revealed that the peel ethanol extract's ethyl acetate layer inhibited urinary bladder urothelial cancer cells more effectively than any other layer. Diaion HP‐20 column chromatography was used to extract the PEPE2 fraction from the ethylacetate layer, which showed an inhibitory effect against bladder urothelial cancer cells. Flow cytometry and apoptotic pathway analysis confirmed that urinary bladder urothelial carcinoma cells cell death was the cause of the PEPE2 fraction's inhibitory impact. The ethyl acetate layer reduced the size and mass of T24 growths and induced apoptosis in the xenografted tumors when administered orally to nude mice with bladder tumors using xenografts. A cost‐effective and attractive chemopreventive product is provided for the transformation of traditionally inedible pomegranate peel in an effort to prevent or mitigate the emergence of urinary bladder urothelial carcinoma cells. The ongoing exploration of such mechanisms could lead to novel strategies for targeting cancer cell proliferation and metastasis in cancers (Chang et al. 2018). The role of punicalagin against different cancers is shown in Table 1.

**TABLE 1:** Punicalagin against different cancers (summarized).

Type of cancer	Study	Intervention	Result	Reference
Breast cancer	In vitro: cytotoxic and apoptotic effects on MCF‐7 cells	(0.2–5.0 g/kg) punicalagin for 72 h	A time‐dependent inhibition of MCF‐7 breast cancer cell proliferation	(Abd‐Rabou et al. 2022)
	In vivo: cytotoxic effect of punicalagin on MDA‐MB‐231 (xenograft mice)	54.21 μg/L. for 24 h	Reduction of tumor cell proliferation in MDA‐MB‐231 and MCF‐7 cancer simulations	(Pan et al. 2020).
	In vivo: DMBA‐induced breast carcinogenesis in rats	(0.2–5.0 g/kg) punicalagin for 72 h	Inhibited DMBA‐induced mammary carcinogenesis	(Mandal et al. 2017).
Pancreatic cancer	In vitro: human pancreatic cancer (Suit‐2)	(10 mg/CAM) for 24 h	Punicalagin attacked PANC‐1 cancer cell lines	Kilit and Aydemir 2023b
Prostate cancer	In vitro: human PC‐3 and LNCaP cells	10, 50, and 100 M of punicalagin for 48 h	Notable decrease in the development of tumor nodules	(Adaramoye et al. 2017).
	In vivo: nude rats	Punicalagin at 50 mg for 12 weeks	Significantly increased apoptotic rate and decreased tumor size	(Ma et al. 2015).
	In vivo: PCa metastasis(severe combined immunodeficiency mouse model)	10 mg/CAM for 1 week	Suppression of signaling pathways	Wang et al. 2014
Colon cancer	In vitro: colorectal cancer cell line HCT 116	Punicalagin at a dose of 5 mg/kg body weight, taken orally for 6 days	Decreased pancreatic cell proliferation in a time‐ and dose‐dependent way	(Kilit and Aydemir 2023b).
	In vivo: anti‐colorectal effects of Punicalagin xenograft mice	24‐h treatment with 100 μM of Punicalagin	Suppressing NF‐κB signaling leads to apoptosis induction and inhibition of cell growth	(Chen et al. 2022)
Bladder cancer	Western blotting analysis of human U2OS	185 μmol for 72 h	Prevents the progression of bladder carcinoma	(Huang et al. 2020).
	In vivo: Sprague Dawley male rats	12 weeks	p53 immunostaining was significantly elevated	Mortada et al. 2020
Oral cancer	HNO‐97 human tongue cancer cell line	Doses of 6.41 μg/ml and 1.21 μg/ml	Significantly lower mean values for BAX expression, caspase‐3 expression, and DNA fragmentation	(Rageh et al. 2023).
	Anti‐angiogenic effects on oral cancer cells	At dosages ranging from 25 and 50 μg/mL for 72‐h exposure	Inhibits the activity of MMP‐2 or MMP‐9	(Imanu et al. 2023)
Liver cancer	Effect of punicalagin on HepG2 cancer cells		Induce apoptosis and boosted apoptotic death	(Li et al. 2019).
	Anti‐proliferative assay on liver carcinoma	24 h of punicalagin exposure at 100 μg/mL	Significantly reduced Bcl‐2 expression levels	(Mehra et al. 2021)
Gastric cancer	In vivo: antiulcerogenic effects of punicalagin in Wistar albino rats	50 mg/kg through a 15‐day oral	Dose‐dependently inhibited the proliferation gastric carcinoma	(Ghazaleh et al. 2013).
	In vivo: antiulcerogenic effects of punicalagin in male rodents	(500 mg/kg)	Significant downregulation of the expression of the TNF‐α	(Mohamed and Mabrok 2021)
Kidney cancer	EMT phenotype in pVHL‐deficient RCCs	50 mg/kg through a 15‐day oral	Inhibiting the NF‐κB and JNK signaling pathways	(Aladaileh et al. 2021)
	Invasion, and proliferation on RCC cells	100 mg kg^−1^ b/w	Inhibition of growth of cancer cells in a dose‐dependent manner and inhibit NF‐κB and JNK	(An et al. 2015).
Blood cancer	In vivo: G0/G1 cell cycle inhibitory effects mouse models	50 mg/kg through a 15‐day oral	Inhibited cell migration and invasion	(Tibullo et al. 2016).
	Cytotoxic effect on U266 cells	24 h of punicalagin exposure at 100 μg/mL	A dose‐dependent reduction in cancer cells was observed	(Kiraz et al. 2016).
	NB4 and MOLT‐4 leukemic cell lines	Punicalagin 1–30 μg/mL for 72‐h	Significantly reduced Bcl‐2 expression levels	(Subkorn et al. 2021).
Brain cancer	Green fluorescence‐LC3 fusion protein method on U87MG cells	(1–30 μg/mL) punicalagin when administered for 24 h	Autophagic cell death	(Wong et al. 2021).
	MTT technique on U87MG glioma cells	Punicalagin 1–30 μg/mL for 72‐h	Influenced cell survival in a dose‐dependent way	(Wang et al. 2013).
	In vitro: anticancer effects on glioma cells	Punicalagin for 24 and 48 h at 0.1, 0.5, and 0.25 mg/mL	Decreased tumor cell viability	(Mota Ferreira et al. 2016).
	In vivo: U87‐MG sphere invasion in a 3D collagen model	Doses of 0.1,0.25, and 0.5 mg/mL over a period of 48 h	Reduced U87‐MG invasion in a manner that depended on both dosage and duration.	Zraikat et al. 2018
Cervical cancer	In vitro: HeLa human cervical carcinoma cells	24, 36, and 48 h with Punicalagin (0, 12.5, 25, 50, 100, and 200 μM).	Block the‐catenin signaling pathway	(Tang et al. 2017).
	ME‐180 cells	Punicalagin 100 μM treatment for 24 h	Blocks NF‐kappa B activity, which in turn limits cervical cancer cell	(Kilit and Aydemir 2023a).
Thyroid cancer	BCPAP thyroid cancer cell line	24‐h treatment with 100 μM of Punicalagin	Inhibited the viability of the BCPAP thyroid cancer cell line	(Cheng et al. 2016)
Lung cancer	In‐vitro: A549 cells	10, 20, and 30 μM Punicalagin for 24 h	Regulates apoptosis, proliferation, and angiogenesis	(Fang et al. 2021)
	Lung epithelial cell line MRC‐5	Punicalagin at concentrations of 50 μM and 75 μM for 1 week	Reduction in cytoplasmic reactive oxygen species (ROS) production	(Kilit and Aydemir 2023a).
	Antiproliferative mechanism on MRC‐5 and A549 cell line	Punicalagin at doses ranging from 1 to 100 mu M For 24 h	Stopped in the G (1)/S phase subsequent	(Berköz and Krosniak 2020)
	In‐vitro: invasion of H1299 cells	10, 20, and 30 μM Punicalagin for 24 h	Non‐toxic and effective chemotherapeutic drug for NSCLC therapy	(Li et al. 2016).

## 5 Conclusion

Punicalagin possesses inhibitory properties against various cancers. A review of the studies and their findings suggests that punicalagin may be an effective treatment or preventive agent for cancers affecting the digestive tract, liver, prostate, breast, and other organs. Punicalagin can prevent the development and replication of cancerous cells using several pathways, as shown in multiple studies. It is lethal to almost every form of malignant/cancerous cell. Punicalagin has the potential to not only inhibit the activity of Nrf2 and the genes under its control but also induce apoptosis in MCF‐7 cancer cells. Punicalagin likely prevents cancer by inhibiting the Akt signaling pathway and generating caspases. Additionally, clinical trials have shown some promise in slowing the progression of cancers and reducing tumor levels, though further studies are necessary to establish the clinical efficacy of pomegranate in prostate cancer treatment. As per the findings, punicalagin treatment inhibits the formation of the Toll‐4 receptor gene (TLR‐4), caspase‐3 protein synthesis, hepatic necrosis, and astrocyte edema. ATC cells undergo apoptosis in response to punicalagin, which inhibits their growth. Its effect is dependent upon both Notch‐1 and SLUG being deactivated simultaneously. Through its ability to inhibit the ROS‐driven Akt/mTOR signaling pathway, punicalagin has the potential to enhance endothelial cell (EC) autophagy, resulting in increased autophagic degradation. Research also shows that the activation of AMPK by punicalagin inhibits Akt, halts the growth of human lung cancer cells, and induces apoptosis. Activation of GPER and reduction in the expressions of ROCK1, TAGLN2, and FCHO2 are some of the processes modulated by punicalagin that inhibit the development of cancer. Utilization of punicalagin stimulated the activity of proteins such as P38, ERK1/2, and mitogen‐activated protein kinases (MAPK) in prostate cancer cells. It can inhibit the activity of phosphoinositide 3‐kinase (PI3K) and reduce the expression of the proteins AKT, P70S6K, S6, and P90RSK. Punicalagin exhibits promise as a pharmaceutical agent due to its ability to inhibit the division of cancer cells, thereby presenting a viable therapeutic option for cancers. Furthermore, these compounds disrupt key molecular pathways like the CXCR4/CXCL12 axis, which is crucial for cancer cell migration and metastasis. Due to the fact that punicalagin inhibits the growth of tumors while remaining non‐toxic and non‐mutating to healthy cells, it may find application in relation to chemotherapeutic agents in the treatment and prevention of cancers. Further research is needed on cellular processes mediated by punicalagin against cancer cells and the effects of the processes in order to ensure its safety and effectiveness as a potential anticancer agent.

## Author Contributions

**Muhammad Hammad Ul Hassan:** conceptualization (equal), writing – original draft (equal). **Muhammad Shahbaz:** conceptualization (equal), writing – original draft (equal). **Ushna Momal:** data curation (equal), methodology (equal). **Hammad Naeem:** writing – review and editing (equal). **Muhammad Imran:** investigation (equal), resources (equal). **Mohamed A. Abdelgawad:** writing – review and editing (equal). **Mohammed M. Ghoneim:** methodology (equal), project administration (equal), validation (equal). **Ehab M. Mostafa:** writing – review and editing (equal). **Ahmed H. El‐Ghorab:** writing – original draft (equal). **Suliman A. Alsagaby:** data curation (equal), methodology (equal), visualization (equal). **Waleed Al Abdulmonem:** conceptualization (equal), validation (equal). **Muzzamal Hussain:** resources (equal), writing – review and editing (equal). **Tadesse Fenta Yehuala:** supervision (equal), visualization (equal).

## Conflicts of Interest

The authors declare no conflicts of interest.

## Data Availability

The data that support the findings of this study are available from the corresponding author upon reasonable request.
